# Supplementary material for: Breast Cancer: How Hippotherapy Bridges the Gap between Healing and Recovery—A Randomized Controlled Clinical Trial
Source: Cancers (Basel). 2023 Feb 19;15(4):1317. doi: 10.3390/cancers15041317 (PMC9953804; doi:10.3390/cancers15041317)
Supplement: Supplementary file 1 [file cancers-15-01317-s001.zip › cancers-2181862-supplementary.pdf]

HippoBreastCa

Prospective two-arm open randomised controlled trial with single-blind analysis for intervention efficacy

CASE REPORT FORM

Version n°1 final 31/01/2017

Patient Number: **Hippo-|___|___|-|___|___|**

INCLUSION VISIT AT THE MIS

DATE OF THE VISIT

Date:	I__I__I / I__I__I / I__I__I__I__I (dd/mm/yyyy)

DATE OF SIGNATURE OF INFORMED CONSENT (DELAY OF 15 DAYS)

Date:	I__I__I / I__I__I / I__I__I__I__I (dd/mm/yyyy)

DEMOGRAPHY

Month and year of birth:<br>Hormonal status:	I__I__I / I__I__I__I__I (mm/yyyy)<br>☐ No-menopausal ☐ Menaupausal
Treatment:	☐ Surgery ☐ Radiotherapy ☐ Chemotherapy ☐ Hormonal therapy

INCLUSION CRITERIA

1. Female gender	☐ Yes ☐ No
2. Age: from 18 to 80 at the time of diagnosis	☐ Yes ☐ No
3. Confirmed histological diagnosis of breast cancer staging [T1-T3, N0-N2 and M0]	☐ Yes ☐ No
4. Patient already scheduled or ongoing treatment for surgery and/or chemotherapy and/or hormone therapy and/or radiotherapy	☐ Yes ☐ No
5. Having consulted a physician of the Care and Support Unit of the MIS during its health care	☐ Yes ☐ No
6. WHO performance index from 0 to 2	☐ Yes ☐ No
7. Able to give her informed consent in writing	☐ Yes ☐ No
8. Able to complete questionnaires	☐ Yes ☐ No
9. Minimal abduction of the hip of 25 degrees bilateral with no history of hip dislocation and/or dysplasia	☐ Yes ☐ No
10. Affiliated to a social security scheme	☐ Yes ☐ No
11. Certificate of no contraindication issued by the responsible physician	☐ Yes ☐ No

NON-INCLUSION CRITERIA

1. History of malignant tumors in the last 5 years	☐ Yes ☐ No
2. Breast cancer as a secondary diagnosis (metastatic)	☐ Yes ☐ No
3. Medication intake or presence of conditions associated with fatigue (e.g. chronic fatigue syndrome)	☐ Yes ☐ No
4. Concomitant and uncontrolled severe degenerative or chronic disease	☐ Yes ☐ No
5. History of allergic reactions to dust and/or horsehair, or asthma	☐ Yes ☐ No
6. Contraindications to physical activity	☐ Yes ☐ No
7. History of horseback riding or hippotherapy treatment during the last 6 months	☐ Yes ☐ No
8. Clinically significant cognitive impairment or dementia	☐ Yes ☐ No
9. Pregnancy and breastfeeding	☐ Yes ☐ No
10. Patient participating in another biomedical research or in exclusion period	☐ Yes ☐ No

If all inclusion criteria are ticked "Yes" and all non-inclusion criteria are ticked "No":

The patient can be included in the study.

RANDOMIZATION

Randomisation number	I___I___I<br><br>Groupe de randomisation: _______________

Please refer to the stratified randomization tables (hormonal status and treatment)

THE PATIENT WILL BE INFORMED OF THE GROUP TO WHICH HE/SHE BELONGS AT THE END OF THE INITIAL ASSESSMENT

FIRST EVALUATION BEFORE THERAPY – BASELINE (D0)

DATE OF COMPLETION OF THE QUESTIONNAIRES

Date:	I__I__I / I__I__I / I__I__I__I__I (dd/mm/yyyy)

PATIENT'S CLINICAL TESTS

European Organization for Research and Treatment of Cancer quality of life questionnaire (EORTC QLQ-C30)

We are interested in some things about you and your health. Please answer all of the questions yourself by circling the number that best applies to you. There are no "right" or "wrong" answers. The information that you provide will remain strictly confidential.

	Not at all	A little	Quite a bit	Very much
1. Do you have any trouble doing strenuous activities, like carrying a heavy shopping bag or a suitcase?	1	2	3	4
2. Do you have any trouble taking a long walk?	1	2	3	4
3. Do you have any trouble taking a short walk outside of the house?	1	2	3	4
4. Do you need to stay in bed or a chair during the day?	1	2	3	4
5. Do you need help with eating, dressing, washing yourself or using the toilet?	1	2	3	4
**During the past week:**				
6. Were you limited in doing either your work or other daily activities?	1	2	3	4
7. Were you limited in pursuing your hobbies or other leisure time activities	1	2	3	4
8. Were you short of breath?	1	2	3	4
9. Have you had pain?	1	2	3	4
10. Did you need to rest?	1	2	3	4
11. Have you had trouble sleeping?	1	2	3	4
12. Have you felt weak?	1	2	3	4
13. Have you lacked appetite?	1	2	3	4
14. Have you felt nauseated?	1	2	3	4
15. Have you vomited?	1	2	3	4
16. Have you been constipated?	1	2	3	4
17. Have you had diarrhea?	1	2	3	4
18. Were you tired?	1	2	3	4

19. Did pain interfere with your daily activities?	1	2	3	4
20. Have you had difficulty in concentrating on things, like reading a newspaper or watching television?	1	2	3	4
21. Did you feel tense?	1	2	3	4
22. Did you worry?	1	2	3	4
23. Did you feel irritable?	1	2	3	4
24. Did you feel depressed?	1	2	3	4
25. Have you had difficulty remembering things?	1	2	3	4
26. Has your physical condition or medical treatment interfered with your family life?	1	2	3	4
27. Has your physical condition or medical treatment interfered with your social activities?	1	2	3	4
28. Has your physical condition or medical treatment caused you financial difficulties?	1	2	3	4

For the following questions please circle the number between 1 and 7 that best applies to you.

29. How would you rate your overall health during the past week?

1 ☐ 2 ☐ 3 ☐ 4 ☐ 5 ☐ 6 ☐ 7 ☐

Very poor Excellent

30. How would you rate your overall quality of life during the past week?

1 ☐ 2 ☐ 3 ☐ 4 ☐ 5 ☐ 6 ☐ 7 ☐

Very poor Excellent

European Organization for Research and Treatment of Cancer quality of life questionnaire (EORTC QLQ-B23)

Patients sometimes report that they have the following symptoms or problems. Please indicate the extent to which you have experienced these symptoms or problems during the past week.

	Not at all	A little	Quite a bit	Very much
**During the past week:**				
31. Did you have a dry mouth?	1	2	3	4
32. Did food and drink taste different than usual?	1	2	3	4
33. Were your eyes painful, irritated or watery?	1	2	3	4
34. Have you lost any hair?	1	2	3	4
35. Answer this question only if you had any hair loss: Were you upset by the loss of your hair?	1	2	3	4
36. Did you feel ill or unwell?	1	2	3	4
37. Did you have hot flushes?	1	2	3	4
38. Did you have headaches?	1	2	3	4
39. Have you felt physically less attractive as a result of your disease or treatment?	1	2	3	4
40. Have you been feeling less feminine as a result of your disease or treatment?	1	2	3	4
41. Did you find it difficult to look at yourself naked?	1	2	3	4
42. Have you been dissatisfied with your body?	1	2	3	4
43. Were you worried about your health in the future?	1	2	3	4
**During the past four weeks:**				
44. To what extent were you interested in sex?	1	2	3	4
45. To what extent were you sexually active? (with or without intercourse)	1	2	3	4
46. Answer this question only if you have been sexually active: To what extent was sex enjoyable for you?	1	2	3	4
**During the past week:**				
47. Did you have any pain in your arm or shoulder?	1	2	3	4
48. Did you have a swollen arm or hand?	1	2	3	4
49. Was it difficult to raise your arm or to move it sideways?	1	2	3	4
50. Have you had any pain in the area of your affected breast?	1	2	3	4
51. Was the area of your affected breast swollen?	1	2	3	4
52. Was the area of your affected breast oversensitive?	1	2	3	4
53. Have you had skin problems on or in the area of your affected breast (e.g., itchy, dry, flaky)?	1	2	3	4

Functional Assessment of Cancer Therapy-Cognitive Function (FACT-Cog)

Below is a list of statements that other people with your condition have said are important. Please circle or mark one number per line to indicate your response as it applies to the past 7 days.

	Never	About once a week	Two to three times a week	Nearly every day	Several times a day
**PERCEIVED COGNITIVE IMPAIRMENTS**					
I have had trouble forming thoughts	0	1	2	3	4
My thinking has been slow	0	1	2	3	4
I have had trouble concentrating	0	1	2	3	4
I have had trouble finding my way to a familiar place	0	1	2	3	4
I have had trouble remembering where I put things, like my keys or my wallet	0	1	2	3	4
I have had trouble remembering new information, like phone numbers or simple instructions	0	1	2	3	4
I have had trouble recalling the name of an object while talking to someone	0	1	2	3	4
I have had trouble finding the right word(s) to express myself	0	1	2	3	4
I have used the wrong word when I referred to an object	0	1	2	3	4
I have had trouble saying what I mean in conversations with others	0	1	2	3	4
I have walked into a room and forgotten what I meant to get or do there	0	1	2	3	4
I have had to work really hard to pay attention or I would make a mistake	0	1	2	3	4
I have forgotten names of people soon after being introduced	0	1	2	3	4
My reactions in everyday situations have been slow	0	1	2	3	4
I have had to work harder than usual to keep track of what I was doing	0	1	2	3	4
My thinking has been slower than usual	0	1	2	3	4
I have had to work harder than usual to express myself clearly	0	1	2	3	4
I have had to use written lists more often than usual so I would not forget things	0	1	2	3	4
I have trouble keeping track of what I am doing if I am interrupted	0	1	2	3	4
I have trouble shifting back and forth between different activities that require thinking	0	1	2	3	4

**COMMENTS FROM OTHERS**

Other people have told me I seemed to have trouble rememberinginformation	0	1	2	3	4
Other people have told me I seemed to have trouble speakingclearly	0	1	2	3	4
Other people have told me I seemed to have trouble thinkingclearly	0	1	2	3	4
Other people have told me I seemed confused	0	1	2	3	4

**PERCEIVED COGNITIVE ABILITIES**					
I have been able to concentrate	0	1	2	3	4
I have been able to bring to mind words that I wanted to use while talking to someone	0	1	2	3	4
I have been able to remember things, like where I left my keys or wallet	0	1	2	3	4
I have been able to remember to do things, like take medicine or buy something I needed	0	1	2	3	4
I am able to pay attention and keep track of what I am doing without extra effort	0	1	2	3	4
My mind is as sharp as it has always been	0	1	2	3	4
My memory is as good as it has always been	0	1	2	3	4
I am able to shift back and forth between two activities that require thinking	0	1	2	3	4
I am able to keep track of what I am doing, even if I am interrupted	0	1	2	3	4

**IMPACT ON QUALITY OF LIFE**					
I have been upset about these problems	0	1	2	3	4
These problems have interfered with my ability to work	0	1	2	3	4
These problems have interfered with my ability to do things I enjoy	0	1	2	3	4
These problems have interfered with the quality of my life	0	1	2	3	4

Multidimensional Fatigue Inventory (MFI-20)

Using the following statements, we would like to understand how you have been feeling recently. Here is an example of a statement: I feel rested. If you think this statement is entirely true, i.e. you agree with it completely, circle the number 5 to the right of the statement, like this:

Answer all questions by circling the appropriate number on the following scale:

1 2 3 4 5

STRONGLY DISAGREE STRONGLY AGREE

	Your answer				
1. I feel fit	1	2	3	4	5
2. Physically, I feel only able to do a little	1	2	3	4	5
3. I feel very active	1	2	3	4	5
4. I feel like doing all sort of nice things	1	2	3	4	5
5. I feel tired	1	2	3	4	5
6. I think I do a lot in a day	1	2	3	4	5
7. When I am doing something, I can keep my thoughts on it	1	2	3	4	5
8. Physically I can take on a lot	1	2	3	4	5
9. I dread having to do things	1	2	3	4	5
10. I think I do very little in a day	1	2	3	4	5
11. I can concentrate well	1	2	3	4	5
12. I am rested	1	2	3	4	5
13. It takes a lot of effort to concentrate on things	1	2	3	4	5
14. Physically I feel I am in a bad condition	1	2	3	4	5
15. I have a lot of plans	1	2	3	4	5
16. I tire easily	1	2	3	4	5
17. I get little done	1	2	3	4	5
18. I don’t feel like doing anything	1	2	3	4	5
19. My thoughts easily wander	1	2	3	4	5
20. Physically I feel I am in an excellent condition	1	2	3	4	5

Hospital Anxiety and Depression Scale (HADS)

Tick the box beside the reply that is closest to how you have been feeling in the past week. Don't take too long over you replies: your immediate is best.

D	A		D	A	
		**I feel tense or 'wound up':**			**I feel as if I am slowed down:**
	3	Most of the time	3		Nearly all the time
	2	A lot of the time	2		Very often
	1	From time to time, occasionally	1		Sometimes
	0	Not at all	0		Not at all
		**I still enjoy the things I used to enjoy:**			**I get a sort of frightened feeling like 'butterflies' in the stomach:**
0		Definitely as much		0	Not at all
1		Not quite so much		1	Occasionally
2		Only a little		2	Quite Often
3		Hardly at all		3	Very Often
		**I get a sort of frightened feeling as if something awful is about to happen:**			**I have lost interest in my appearance:**
	3	Very definitely and quite badly	3		Definitely
	2	Yes, but not too badly	2		I don't take as much care as I should
	1	A little, but it doesn't worry me	1		I may not take quite as much care
	0	Not at all	0		I take just as much care as ever
		**I can laugh and see the funny side of things:**			**I feel restless as I have to be on the move:**
0		As much as I always could		3	Very much indeed
1		Not quite so much now		2	Quite a lot
2		Definitely not so much now		1	Not very much
3		Not at all		0	Not at all
		**Worrying thoughts go through my mind:**			**I look forward with enjoyment to things:**
	3	A great deal of the time	0		As much as I ever did
	2	A lot of the time	1		Rather less than I used to
	1	From time to time, but not too often	2		Definitely less than I used to
	0	Only occasionally	3		Hardly at all
		**I feel cheerful:**			**I get sudden feelings of panic:**
3		Not at all		3	Very often indeed
2		Not often		2	Quite often
1		Sometimes		1	Not very often
0		Most of the time		0	Not at all
		**I can sit at ease and feel relaxed:**			**I can enjoy a good book or radio or TV program:**
	0	Definitely	0		Often
	1	Usually	1		Sometimes
	2	Not Often	2		Not often
	3	Not at all	3		Very seldom

Body Image Scale (BIS)

This questionnaire asks you to tell us how you feel about your physical appearance and the changes caused by your illness or its treatment. Please read each question carefully and circle the answer that best describes how you feel over the past week.

	Not at all	A little	Quite a bit	Very much
1. Did you feel self-conscious about your appearance?	1	2	3	4
2. Have you felt physically less attractive as a result of the disease or treatment?	1	2	3	4
3. Have you been dissatisfied with your appearance when dressed?	1	2	3	4
4. Have you been feeling less feminine/masculine as a result of your disease or treatment?	1	2	3	4
5. Do you find it difficult to look at yourself naked?	1	2	3	4
6. have you been feeling less sexually attractive as a result of your disease or treatment?	1	2	3	4
7. Do you avoid people because of the way you feel about your appearance?	1	2	3	4
8. Did you feel the disease or treatment has left your body less whole?	1	2	3	4
9. Have you felt dissatisfied with your body?	1	2	3	4

End of the visit

SECOND EVALUATION – FIRST POST-TREATMENT EVALUATION (AFTER 1 WEEK)

DATE OF COMPLETION OF THE QUESTIONNAIRES

Date:	I__I__I / I__I__I / I__I__I__I__I (dd/mm/yyyy)

PATIENT'S CLINICAL TESTS

European Organization for Research and Treatment of Cancer quality of life questionnaire (EORTC QLQ-C30)

We are interested in some things about you and your health. Please answer all of the questions yourself by circling the number that best applies to you. There are no "right" or "wrong" answers. The information that you provide will remain strictly confidential.

	Not at all	A little	Quite a bit	Very much
1. Do you have any trouble doing strenuous activities, like carrying a heavy shopping bag or a suitcase?	1	2	3	4
2. Do you have any trouble taking a long walk?	1	2	3	4
3. Do you have any trouble taking a short walk outside of the house?	1	2	3	4
4. Do you need to stay in bed or a chair during the day?	1	2	3	4
5. Do you need help with eating, dressing, washing yourself or using the toilet?	1	2	3	4
**During the past week:**				
6. Were you limited in doing either your work or other daily activities?	1	2	3	4
7. Were you limited in pursuing your hobbies or other leisure time activities	1	2	3	4
8. Were you short of breath?	1	2	3	4
9. Have you had pain?	1	2	3	4
10. Did you need to rest?	1	2	3	4
11. Have you had trouble sleeping?	1	2	3	4
12. Have you felt weak?	1	2	3	4
13. Have you lacked appetite?	1	2	3	4
14. Have you felt nauseated?	1	2	3	4
15. Have you vomited?	1	2	3	4
16. Have you been constipated?	1	2	3	4
17. Have you had diarrhea?	1	2	3	4

18. Were you tired?	1	2	3	4
19. Did pain interfere with your daily activities?	1	2	3	4
20. Have you had difficulty in concentrating on things, like reading a newspaper or watching television?	1	2	3	4
21. Did you feel tense?	1	2	3	4
22. Did you worry?	1	2	3	4
23. Did you feel irritable?	1	2	3	4
24. Did you feel depressed?	1	2	3	4
25. Have you had difficulty remembering things?	1	2	3	4
26. Has your physical condition or medical treatment interfered with your family life?	1	2	3	4
27. Has your physical condition or medical treatment interfered with your social activities?	1	2	3	4
28. Has your physical condition or medical treatment caused you financial difficulties?	1	2	3	4

For the following questions please circle the number between 1 and 7 that best applies to you.

29. How would you rate your overall health during the past week?

1 ☐ 2 ☐ 3 ☐ 4 ☐ 5 ☐ 6 ☐ 7 ☐

Very poor Excellent

30. How would you rate your overall quality of life during the past week?

1 ☐ 2 ☐ 3 ☐ 4 ☐ 5 ☐ 6 ☐ 7 ☐

Very poor Excellent

European Organization for Research and Treatment of Cancer quality of life questionnaire (EORTC QLQ-B23)

Patients sometimes report that they have the following symptoms or problems. Please indicate the extent to which you have experienced these symptoms or problems during the past week.

	Not at all	A little	Quite a bit	Very much
**During the past week:**				
31. Did you have a dry mouth?	1	2	3	4
32. Did food and drink taste different than usual?	1	2	3	4
33. Were your eyes painful, irritated or watery?	1	2	3	4
34. Have you lost any hair?	1	2	3	4
35. Answer this question only if you had any hair loss: Were you upset by the loss of your hair?	1	2	3	4
36. Did you feel ill or unwell?	1	2	3	4
37. Did you have hot flushes?	1	2	3	4
38. Did you have headaches?	1	2	3	4
39. Have you felt physically less attractive as a result of your disease or treatment?	1	2	3	4
40. Have you been feeling less feminine as a result of your disease or treatment?	1	2	3	4
41. Did you find it difficult to look at yourself naked?	1	2	3	4
42. Have you been dissatisfied with your body?	1	2	3	4
43. Were you worried about your health in the future?	1	2	3	4
**During the past four weeks:**				
44. To what extent were you interested in sex?	1	2	3	4
45. To what extent were you sexually active? (with or without intercourse)	1	2	3	4
46. Answer this question only if you have been sexually active: To what extent was sex enjoyable for you?	1	2	3	4
**During the past week:**				
47. Did you have any pain in your arm or shoulder?	1	2	3	4
48. Did you have a swollen arm or hand?	1	2	3	4
49. Was it difficult to raise your arm or to move it sideways?	1	2	3	4
50. Have you had any pain in the area of your affected breast?	1	2	3	4
51. Was the area of your affected breast swollen?	1	2	3	4
52. Was the area of your affected breast oversensitive?	1	2	3	4
53. Have you had skin problems on or in the area of your affected breast (e.g., itchy, dry, flaky)?	1	2	3	4

Functional Assessment of Cancer Therapy-Cognitive Function (FACT-Cog)

Below is a list of statements that other people with your condition have said are important. Please circle or mark one number per line to indicate your response as it applies to the past 7 days.

	Never	About once a week	Two to three times a week	Nearly every day	Several times a day
**PERCEIVED COGNITIVE IMPAIRMENTS**					
I have had trouble forming thoughts	0	1	2	3	4
My thinking has been slow	0	1	2	3	4
I have had trouble concentrating	0	1	2	3	4
I have had trouble finding my way to a familiar place	0	1	2	3	4
I have had trouble remembering where I put things, like my keys or my wallet	0	1	2	3	4
I have had trouble remembering new information, like phone numbers or simple instructions	0	1	2	3	4
I have had trouble recalling the name of an object while talking to someone	0	1	2	3	4
I have had trouble finding the right word(s) to express myself	0	1	2	3	4
I have used the wrong word when I referred to an object	0	1	2	3	4
I have had trouble saying what I mean in conversations with others	0	1	2	3	4
I have walked into a room and forgotten what I meant to get or do there	0	1	2	3	4
I have had to work really hard to pay attention or I would make a mistake	0	1	2	3	4
I have forgotten names of people soon after being introduced	0	1	2	3	4
My reactions in everyday situations have been slow	0	1	2	3	4
I have had to work harder than usual to keep track of what I was doing	0	1	2	3	4
My thinking has been slower than usual	0	1	2	3	4
I have had to work harder than usual to express myself clearly	0	1	2	3	4
I have had to use written lists more often than usual so I would not forget things	0	1	2	3	4
I have trouble keeping track of what I am doing if I am interrupted	0	1	2	3	4
I have trouble shifting back and forth between different activities that require thinking	0	1	2	3	4

COMMENTS FROM OTHERS

Other people have told me I seemed to have trouble rememberinginformation	0	1	2	3	4
Other people have told me I seemed to have trouble speakingclearly	0	1	2	3	4
Other people have told me I seemed to have trouble thinkingclearly	0	1	2	3	4
Other people have told me I seemed confused	0	1	2	3	4

**PERCEIVED COGNITIVE ABILITIES**					
I have been able to concentrate	0	1	2	3	4
I have been able to bring to mind words that I wanted to use while talking to someone	0	1	2	3	4
I have been able to remember things, like where I left my keys or wallet	0	1	2	3	4
I have been able to remember to do things, like take medicine or buy something I needed	0	1	2	3	4
I am able to pay attention and keep track of what I am doing without extra effort	0	1	2	3	4
My mind is as sharp as it has always been	0	1	2	3	4
My memory is as good as it has always been	0	1	2	3	4
I am able to shift back and forth between two activities that require thinking	0	1	2	3	4
I am able to keep track of what I am doing, even if I am interrupted	0	1	2	3	4

**IMPACT ON QUALITY OF LIFE**					
I have been upset about these problems	0	1	2	3	4
These problems have interfered with my ability to work	0	1	2	3	4
These problems have interfered with my ability to do things I enjoy	0	1	2	3	4
These problems have interfered with the quality of my life	0	1	2	3	4

Multidimensional Fatigue Inventory (MFI-20)

Using the following statements, we would like to understand how you have been feeling recently. Here is an example of a statement: I feel rested. If you think this statement is entirely true, i.e. you agree with it completely, circle the number 5 to the right of the statement, like this:

Answer all questions by circling the appropriate number on the following scale:

1 2 3 4 5

STRONGLY DISAGREE STRONGLY AGREE

	**Your answer**				
1. I feel fit	1	2	3	4	5
2. Physically, I feel only able to do a little	1	2	3	4	5
3. I feel very active	1	2	3	4	5
4. I feel like doing all sort of nice things	1	2	3	4	5
5. I feel tired	1	2	3	4	5
6. I think I do a lot in a day	1	2	3	4	5
7. When I am doing something, I can keep my thoughts on it	1	2	3	4	5
8. Physically I can take on a lot	1	2	3	4	5
9. I dread having to do things	1	2	3	4	5
10. I think I do very little in a day	1	2	3	4	5
11. I can concentrate well	1	2	3	4	5
12. I am rested	1	2	3	4	5
13. It takes a lot of effort to concentrate on things	1	2	3	4	5
14. Physically I feel I am in a bad condition	1	2	3	4	5
15. I have a lot of plans	1	2	3	4	5
16. I tire easily	1	2	3	4	5
17. I get little done	1	2	3	4	5
18. I don’t feel like doing anything	1	2	3	4	5
19. My thoughts easily wander	1	2	3	4	5
20. Physically I feel I am in an excellent condition	1	2	3	4	5

Hospital Anxiety and Depression Scale (HADS)

Tick the box beside the reply that is closest to how you have been feeling in the past week. Don't take too long over you replies: your immediate is best.

D	A		D	A	
		**I feel tense or 'wound up':**			**I feel as if I am slowed down:**
	3	Most of the time	3		Nearly all the time
	2	A lot of the time	2		Very often
	1	From time to time, occasionally	1		Sometimes
	0	Not at all	0		Not at all
		**I still enjoy the things I used to enjoy:**			**I get a sort of frightened feeling like 'butterflies' in the stomach:**
0		Definitely as much		0	Not at all
1		Not quite so much		1	Occasionally
2		Only a little		2	Quite Often
3		Hardly at all		3	Very Often
		**I get a sort of frightened feeling as if something awful is about to happen:**			**I have lost interest in my appearance:**
	3	Very definitely and quite badly	3		Definitely
	2	Yes, but not too badly	2		I don't take as much care as I should
	1	A little, but it doesn't worry me	1		I may not take quite as much care
	0	Not at all	0		I take just as much care as ever
		**I can laugh and see the funny side of things:**			**I feel restless as I have to be on the move:**
0		As much as I always could		3	Very much indeed
1		Not quite so much now		2	Quite a lot
2		Definitely not so much now		1	Not very much
3		Not at all		0	Not at all
		**Worrying thoughts go through my mind:**			**I look forward with enjoyment to things:**
	3	A great deal of the time	0		As much as I ever did
	2	A lot of the time	1		Rather less than I used to
	1	From time to time, but not too often	2		Definitely less than I used to
	0	Only occasionally	3		Hardly at all
		**I feel cheerful:**			**I get sudden feelings of panic:**
3		Not at all		3	Very often indeed
2		Not often		2	Quite often
1		Sometimes		1	Not very often
0		Most of the time		0	Not at all
		**I can sit at ease and feel relaxed:**			**I can enjoy a good book or radio or TV program:**
	0	Definitely	0		Often
	1	Usually	1		Sometimes
	2	Not Often	2		Not often
	3	Not at all	3		Very seldom

Body Image Scale (BIS)

This questionnaire asks you to tell us how you feel about your physical appearance and the changes caused by your illness or its treatment. Please read each question carefully and circle the answer that best describes how you feel over the past week.

	Not at all	A little	Quite a bit	Very much
1. Did you feel self-conscious about your appearance?	1	2	3	4
2. Have you felt physically less attractive as a result of the disease or treatment?	1	2	3	4
3. Have you been dissatisfied with your appearance when dressed?	1	2	3	4
4. Have you been feeling less feminine/masculine as a result of your disease or treatment?	1	2	3	4
5. Do you find it difficult to look at yourself naked?	1	2	3	4
6. have you been feeling less sexually attractive as a result of your disease or treatment?	1	2	3	4
7. Do you avoid people because of the way you feel about your appearance?	1	2	3	4
8. Did you feel the disease or treatment has left your body less whole?	1	2	3	4
9. Have you felt dissatisfied with your body?	1	2	3	4

End of the visit

THIRD EVALUATION – FINAL POST-TREATMENT EVALUATION (AFTER 6 MOTHS)

DATE OF COMPLETION OF THE QUESTIONNAIRES

Date:	I__I__I / I__I__I / I__I__I__I__I (dd/mm/yyyy)

PATIENT'S CLINICAL TESTS

European Organization for Research and Treatment of Cancer quality of life questionnaire (EORTC QLQ-C30)

We are interested in some things about you and your health. Please answer all of the questions yourself by circling the number that best applies to you. There are no "right" or "wrong" answers. The information that you provide will remain strictly confidential.

	Not at all	A little	Quite a bit	Very much
1. Do you have any trouble doing strenuous activities, like carrying a heavy shopping bag or a suitcase?	1	2	3	4
2. Do you have any trouble taking a long walk?	1	2	3	4
3. Do you have any trouble taking a short walk outside of the house?	1	2	3	4
4. Do you need to stay in bed or a chair during the day?	1	2	3	4
5. Do you need help with eating, dressing, washing yourself or using the toilet?	1	2	3	4
**During the past week:**				
6. Were you limited in doing either your work or other daily activities?	1	2	3	4
7. Were you limited in pursuing your hobbies or other leisure time activities	1	2	3	4
8. Were you short of breath?	1	2	3	4
9. Have you had pain?	1	2	3	4
10. Did you need to rest?	1	2	3	4
11. Have you had trouble sleeping?	1	2	3	4
12. Have you felt weak?	1	2	3	4
13. Have you lacked appetite?	1	2	3	4
14. Have you felt nauseated?	1	2	3	4
15. Have you vomited?	1	2	3	4
16. Have you been constipated?	1	2	3	4
17. Have you had diarrhea?	1	2	3	4

18. Were you tired?	1	2	3	4
19. Did pain interfere with your daily activities?	1	2	3	4
20. Have you had difficulty in concentrating on things, like reading a newspaper or watching television?	1	2	3	4
21. Did you feel tense?	1	2	3	4
22. Did you worry?	1	2	3	4
23. Did you feel irritable?	1	2	3	4
24. Did you feel depressed?	1	2	3	4
25. Have you had difficulty remembering things?	1	2	3	4
26. Has your physical condition or medical treatment interfered with your family life?	1	2	3	4
27. Has your physical condition or medical treatment interfered with your social activities?	1	2	3	4
28. Has your physical condition or medical treatment caused you financial difficulties?	1	2	3	4

For the following questions please circle the number between 1 and 7 that best applies to you.

29. How would you rate your overall health during the past week?

1 ☐ 2 ☐ 3 ☐ 4 ☐ 5 ☐ 6 ☐ 7 ☐

Very poor Excellent

30. How would you rate your overall quality of life during the past week?

1 ☐ 2 ☐ 3 ☐ 4 ☐ 5 ☐ 6 ☐ 7 ☐

Very poor Excellent

European Organization for Research and Treatment of Cancer quality of life questionnaire (EORTC QLQ-B23)

Patients sometimes report that they have the following symptoms or problems. Please indicate the extent to which you have experienced these symptoms or problems during the past week.

	Not at all	A little	Quite a bit	Very much
**During the past week:**				
31. Did you have a dry mouth?	1	2	3	4
32. Did food and drink taste different than usual?	1	2	3	4
33. Were your eyes painful, irritated or watery?	1	2	3	4
34. Have you lost any hair?	1	2	3	4
35. Answer this question only if you had any hair loss: Were you upset by the loss of your hair?	1	2	3	4
36. Did you feel ill or unwell?	1	2	3	4
37. Did you have hot flushes?	1	2	3	4
38. Did you have headaches?	1	2	3	4
39. Have you felt physically less attractive as a result of your disease or treatment?	1	2	3	4
40. Have you been feeling less feminine as a result of your disease or treatment?	1	2	3	4
41. Did you find it difficult to look at yourself naked?	1	2	3	4
42. Have you been dissatisfied with your body?	1	2	3	4
43. Were you worried about your health in the future?	1	2	3	4
**During the past four weeks:**				
44. To what extent were you interested in sex?	1	2	3	4
45. To what extent were you sexually active? (with or without intercourse)	1	2	3	4
46. Answer this question only if you have been sexually active: To what extent was sex enjoyable for you?	1	2	3	4
**During the past week:**				
47. Did you have any pain in your arm or shoulder?	1	2	3	4
48. Did you have a swollen arm or hand?	1	2	3	4
49. Was it difficult to raise your arm or to move it sideways?	1	2	3	4
50. Have you had any pain in the area of your affected breast?	1	2	3	4
51. Was the area of your affected breast swollen?	1	2	3	4
52. Was the area of your affected breast oversensitive?	1	2	3	4
53. Have you had skin problems on or in the area of your affected breast (e.g., itchy, dry, flaky)?	1	2	3	4

Functional Assessment of Cancer Therapy-Cognitive Function (FACT-Cog)

Below is a list of statements that other people with your condition have said are important. Please circle or mark one number per line to indicate your response as it applies to the past 7 days.

	Never	About once a week	Two to three times a week	Nearly every day	Several times a day
**PERCEIVED COGNITIVE IMPAIRMENTS**					
I have had trouble forming thoughts	0	1	2	3	4
My thinking has been slow	0	1	2	3	4
I have had trouble concentrating	0	1	2	3	4
I have had trouble finding my way to a familiar place	0	1	2	3	4
I have had trouble remembering where I put things, like my keys or my wallet	0	1	2	3	4
I have had trouble remembering new information, like phone numbers or simple instructions	0	1	2	3	4
I have had trouble recalling the name of an object while talking to someone	0	1	2	3	4
I have had trouble finding the right word(s) to express myself	0	1	2	3	4
I have used the wrong word when I referred to an object	0	1	2	3	4
I have had trouble saying what I mean in conversations with others	0	1	2	3	4
I have walked into a room and forgotten what I meant to get or do there	0	1	2	3	4
I have had to work really hard to pay attention or I would make a mistake	0	1	2	3	4
I have forgotten names of people soon after being introduced	0	1	2	3	4
My reactions in everyday situations have been slow	0	1	2	3	4
I have had to work harder than usual to keep track of what I was doing	0	1	2	3	4
My thinking has been slower than usual	0	1	2	3	4
I have had to work harder than usual to express myself clearly	0	1	2	3	4
I have had to use written lists more often than usual so I would not forget things	0	1	2	3	4
I have trouble keeping track of what I am doing if I am interrupted	0	1	2	3	4
I have trouble shifting back and forth between different activities that require thinking	0	1	2	3	4

COMMENTS FROM OTHERS

Other people have told me I seemed to have trouble rememberinginformation	0	1	2	3	4
Other people have told me I seemed to have trouble speakingclearly	0	1	2	3	4
Other people have told me I seemed to have trouble thinkingclearly	0	1	2	3	4
Other people have told me I seemed confused	0	1	2	3	4

**PERCEIVED COGNITIVE ABILITIES**					
I have been able to concentrate	0	1	2	3	4
I have been able to bring to mind words that I wanted to use while talking to someone	0	1	2	3	4
I have been able to remember things, like where I left my keys or wallet	0	1	2	3	4
I have been able to remember to do things, like take medicine or buy something I needed	0	1	2	3	4
I am able to pay attention and keep track of what I am doing without extra effort	0	1	2	3	4
My mind is as sharp as it has always been	0	1	2	3	4
My memory is as good as it has always been	0	1	2	3	4
I am able to shift back and forth between two activities that require thinking	0	1	2	3	4
I am able to keep track of what I am doing, even if I am interrupted	0	1	2	3	4

**IMPACT ON QUALITY OF LIFE**					
I have been upset about these problems	0	1	2	3	4
These problems have interfered with my ability to work	0	1	2	3	4
These problems have interfered with my ability to do things I enjoy	0	1	2	3	4
These problems have interfered with the quality of my life	0	1	2	3	4

Multidimensional Fatigue Inventory (MFI-20)

Using the following statements, we would like to understand how you have been feeling recently. Here is an example of a statement: I feel rested. If you think this statement is entirely true, i.e. you agree with it completely, circle the number 5 to the right of the statement, like this:

Answer all questions by circling the appropriate number on the following scale:

1 2 3 4 5

STRONGLY DISAGREE STRONGLY AGREE

	**Your answer**				
1. I feel fit	1	2	3	4	5
2. Physically, I feel only able to do a little	1	2	3	4	5
3. I feel very active	1	2	3	4	5
4. I feel like doing all sort of nice things	1	2	3	4	5
5. I feel tired	1	2	3	4	5
6. I think I do a lot in a day	1	2	3	4	5
7. When I am doing something, I can keep my thoughts on it	1	2	3	4	5
8. Physically I can take on a lot	1	2	3	4	5
9. I dread having to do things	1	2	3	4	5
10. I think I do very little in a day	1	2	3	4	5
11. I can concentrate well	1	2	3	4	5
12. I am rested	1	2	3	4	5
13. It takes a lot of effort to concentrate on things	1	2	3	4	5
14. Physically I feel I am in a bad condition	1	2	3	4	5
15. I have a lot of plans	1	2	3	4	5
16. I tire easily	1	2	3	4	5
17. I get little done	1	2	3	4	5
18. I don't feel like doing anything	1	2	3	4	5
19. My thoughts easily wander	1	2	3	4	5
20. Physically I feel I am in an excellent condition	1	2	3	4	5

Hospital Anxiety and Depression Scale (HADS)

Tick the box beside the reply that is closest to how you have been feeling in the past week. Don't take too long over you replies: your immediate is best.

D	A		D	A	
		**I feel tense or 'wound up':**			**I feel as if I am slowed down:**
	3	Most of the time	3		Nearly all the time
	2	A lot of the time	2		Very often
	1	From time to time, occasionally	1		Sometimes
	0	Not at all	0		Not at all
		**I still enjoy the things I used to enjoy:**			**I get a sort of frightened feeling like 'butterflies' in the stomach:**
0		Definitely as much		0	Not at all
1		Not quite so much		1	Occasionally
2		Only a little		2	Quite Often
3		Hardly at all		3	Very Often
		**I get a sort of frightened feeling as if something awful is about to happen:**			**I have lost interest in my appearance:**
	3	Very definitely and quite badly	3		Definitely
	2	Yes, but not too badly	2		I don't take as much care as I should
	1	A little, but it doesn't worry me	1		I may not take quite as much care
	0	Not at all	0		I take just as much care as ever
		**I can laugh and see the funny side of things:**			**I feel restless as I have to be on the move:**
0		As much as I always could		3	Very much indeed
1		Not quite so much now		2	Quite a lot
2		Definitely not so much now		1	Not very much
3		Not at all		0	Not at all
		**Worrying thoughts go through my mind:**			**I look forward with enjoyment to things:**
	3	A great deal of the time	0		As much as I ever did
	2	A lot of the time	1		Rather less than I used to
	1	From time to time, but not too often	2		Definitely less than I used to
	0	Only occasionally	3		Hardly at all
		**I feel cheerful:**			**I get sudden feelings of panic:**
3		Not at all		3	Very often indeed
2		Not often		2	Quite often
1		Sometimes		1	Not very often
0		Most of the time		0	Not at all
		**I can sit at ease and feel relaxed:**			**I can enjoy a good book or radio or TV program:**
	0	Definitely	0		Often
	1	Usually	1		Sometimes
	2	Not Often	2		Not often
	3	Not at all	3		Very seldom

Body Image Scale (BIS)

This questionnaire asks you to tell us how you feel about your physical appearance and the changes caused by your illness or its treatment. Please read each question carefully and circle the answer that best describes how you feel over the past week.

	Not at all	A little	Quite a bit	Very much
1. Did you feel self-conscious about your appearance?	1	2	3	4
2. Have you felt physically less attractive as a result of the disease or treatment?	1	2	3	4
3. Have you been dissatisfied with your appearance when dressed?	1	2	3	4
4. Have you been feeling less feminine/masculine as a result of your disease or treatment?	1	2	3	4
5. Do you find it difficult to look at yourself naked?	1	2	3	4
6. have you been feeling less sexually attractive as a result of your disease or treatment?	1	2	3	4
7. Do you avoid people because of the way you feel about your appearance?	1	2	3	4
8. Did you feel the disease or treatment has left your body less whole?	1	2	3	4
9. Have you felt dissatisfied with your body?	1	2	3	4

End of the visit

EQUIPHORIA	Patient number **Hippo-I___I___I-I___I___I**	**HippoBreastCa**

REPORT STAY NO. 1 EQUIPHORIA

(first program, 1 week)

START DATE OF STAY

Date:	I__I__I / I__I__I / I__I__I__I__I (dd/mm/yyyy)

END DATE OF STAY

Date:	I__I__I / I__I__I / I__I__I__I__I (dd/mm/yyyy)

Has the patient completed all the hippotherapy sessions in the centre?	☐ Yes ☐ No
If no, how many sessions did she did?	I__I / 5<br>Done Planned
If no, what were the reasons for not being able to complete the sessions?	☐ Patient refusal<br>☐ Patient's health status<br>*If this box is ticked, please complete the adverse event form*<br>☐ Other<br>Specify: ______________________________

REPORT STAY NO. 2 EQUIPHORIA

(second program, 2 days)

START DATE OF STAY

Date:	I__I__I / I__I__I / I__I__I__I__I (dd/mm/yyyy)

END DATE OF STAY

Date:	I__I__I / I__I__I / I__I__I__I__I (dd/mm/yyyy)

Has the patient completed all the hippotherapy sessions in the centre?	☐ Yes ☐ No
If no, how many sessions did she did?	I__I / 2<br>Done Planned
If no, what were the reasons for not being able to complete the sessions?	☐ Patient refusal<br>☐ Patient's health status<br>*If this box is ticked, please complete the adverse event form*<br>☐ Other<br>Specify: ______________________________

REPORT STAY NO. 3 EQUIPHORIA

(third program, 2 days)

START DATE OF STAY

Date:	I__I__I / I__I__I / I__I__I__I__I (dd/mm/yyyy)

END DATE OF STAY

Date:	I__I__I / I__I__I / I__I__I__I__I (dd/mm/yyyy)

Has the patient completed all the hippotherapy sessions in the centre?	☐ Yes ☐ No
If no, how many sessions did she did?	I__I / 2<br>Done Planned
If no, what were the reasons for not being able to complete the sessions?	☐ Patient refusal<br>☐ Patient's health status<br>*If this box is ticked, please complete the adverse event form*<br>☐ Other<br>Specify: ______________________________

REPORT STAY NO. 4 EQUIPHORIA

(fourth program, 2 days)

START DATE OF STAY

Date:	I__I__I / I__I__I / I__I__I__I__I (dd/mm/yyyy)

END DATE OF STAY

Date:	I__I__I / I__I__I / I__I__I__I__I (dd/mm/yyyy)

Has the patient completed all the hippotherapy sessions in the centre?	☐ Yes ☐ No
If no, how many sessions did she did?	I__I / 2<br>Done Planned
If no, what were the reasons for not being able to complete the sessions?	☐ Patient refusal<br>☐ Patient's health status<br>*If this box is ticked, please complete the adverse event form*<br>☐ Other<br>Specify: ______________________________

END OF STUDY

END DATE

Date:	|__|__| / |__|__| / |__|__|__|__| (dd/mm/yyyy)

MAIN REASON FOR STOPPING THE STUDY

☐	Completed protocol
☐	Adverse event (AE N° |__|__|)
☐	Lost to follow-up
☐	Patient's decision, please give the reason given by the patient: ______________________________
☐	Center's decision, please specify: ________________________________
☐	Doctor's decision, please specify: ________________________________
☐	No-compliance on Equiphoria sessions (no respect of the protocol)

TREATMENT DISCONTINUATION FORM

DATE OF CESSATION OF TREATMENT?

Date:	|__|__| / |__|__| / |__|__|__|__| (dd/mm/yyyy)

Number of visits	|__|__|

Please indicate the reason(s) why the treatment under study was stopped:	
The patient has withdrawn consent	☐ Yes ☐ No
Adverse event	☐ Yes ☐ No<br>*If YES, please complete the AE form*
Pregnancy	☐ Yes ☐ No<br>*If YES, please complete the pregnancy form*
Deaths	☐ Yes ☐ No<br>*If YES, please complete the D form*
Other, please specify: ________________________________	☐ Yes ☐ No
